# Priority effects dictate community structure and alter virulence of fungal-bacterial biofilms

**DOI:** 10.1038/s41396-021-00901-5

**Published:** 2021-02-08

**Authors:** J. Z. Alex Cheong, Chad J. Johnson, Hanxiao Wan, Aiping Liu, John F. Kernien, Angela L. F. Gibson, Jeniel E. Nett, Lindsay R. Kalan

**Affiliations:** 1 Department of Medical Microbiology and Immunology, University of Wisconsin–Madison, School of Medicine and Public Health, Madison, WI USA; 2 Department of Medicine, Division of Infectious Disease, University of Wisconsin–Madison, School of Medicine and Public Health, Madison, WI USA; 3 Department of Surgery, University of Wisconsin–Madison, School of Medicine and Public Health, Madison, WI USA

**Keywords:** Fungi, Biofilms, Microbial ecology, Pathogenesis

## Abstract

Polymicrobial biofilms are a hallmark of chronic wound infection. The forces governing assembly and maturation of these microbial ecosystems are largely unexplored but the consequences on host response and clinical outcome can be significant. In the context of wound healing, formation of a biofilm and a stable microbial community structure is associated with impaired tissue repair resulting in a non-healing chronic wound. These types of wounds can persist for years simmering below the threshold of classically defined clinical infection (which includes heat, pain, redness, and swelling) and cycling through phases of recurrent infection. In the most severe outcome, amputation of lower extremities may occur if spreading infection ensues. Here we take an ecological perspective to study priority effects and competitive exclusion on overall biofilm community structure in a three-membered community comprised of strains of *Staphylococcus aureus, Citrobacter freundii*, and *Candida albicans* derived from a chronic wound. We show that both priority effects and inter-bacterial competition for binding to *C. albicans* biofilms significantly shape community structure on both abiotic and biotic substrates, such as ex vivo human skin wounds. We further show attachment of *C. freundii* to *C. albicans* is mediated by mannose-binding lectins. Co-cultures of *C. freundii* and *C. albicans* trigger the yeast-to-hyphae transition, resulting in a significant increase in neutrophil death and inflammation compared to either species alone. Collectively, the results presented here facilitate our understanding of fungal-bacterial interactions and their effects on host-microbe interactions, pathogenesis, and ultimately, wound healing.

## Introduction

Diverse microbial communities colonize nearly every ecosystem across the human body. Within specific niches, microbe-microbe interactions can play a significant role in driving community assembly and subsequent structural and functional properties. However, the forces governing these processes within the context of tissue microenvironment and host responses are largely undefined. Although a diverse microbiome is often associated with human health [1], chronic wounds frequently harbor diverse microbial communities. An archetypal example is the diabetic foot ulcer (DFU). The development of DFUs can be attributed to numerous host-associated factors such as hyperglycemia, vascular disease, and neuropathy [2–5] leading to the colonization and assembly of a distinct and diverse wound microbiome within the tissue, often without clinical signs of infection [6–16]. The wound microbiome is hypothesized to exist as a polymicrobial biofilm and it has been shown that up to 60% of all chronic wounds contain a biofilm [17–19]. Up to 25% of all persons with diabetes will develop a DFU in their lifetime [20] equating to ~9 million people in the United States alone. Beyond the staggering healthcare costs of up to $19 billion per year, the 5-year mortality rate is between 43 and 55% and increases to 74% if an amputation occurs [21–25].

Longitudinal studies in DFU patients have demonstrated that microbial community stability, or less change over time, is associated with worse wound healing outcomes [6, 12, 14, 26]. The majority of these studies have focused on bacteria, yet fungi have been reported to be present alongside diverse bacterial communities in up to 75% of DFUs [8, 26, 27]. The presence of fungi within these communities has been shown to be associated with poorer wound outcomes and higher amounts of necrosis or dead tissue [26], suggesting that antifungal treatment may be beneficial. Thus, fungal-bacterial infection can complicate DFU treatment by requiring both antifungal and antibacterial antibiotics [27, 28]. Furthermore, both bacteria and fungi from wounds are reported to be underestimated via standard culture-based methods [7, 26], presenting an obstacle to effective diagnosis and targeted treatment. Cross-kingdom fungal-bacterial interactions are of interest as they may be critical in shaping microbial community structure and effects on physiology, pathogenesis, and host responses [29–32].

The most common fungal and bacterial species detected in DFU are *Candida albicans* and *Staphylococcus aureus*, found in 47 and 95% of DFUs respectively [6–8, 13, 15, 16, 26, 33]. Interactions between these species are synergistic and enabled via cell-cell adhesion and cross-feeding mechanisms [34–36]. Attachment of *S. aureus* to *C. albicans* in biofilms is well studied [37, 38] and serve as model for studying cross-kingdom interactions [39–42]. These data further suggest that fungi may act as keystone species that can stabilize microbial communities by providing physical scaffolding for bacterial attachment and growth [43–45]. Such networks can be highly complex and dependent on microbe-microbe interactions. For example, the Gram-negative bacterium *Pseudomonas aeruginosa* can have both synergistic and antagonistic effects on *C. albicans*, even resulting in fungal death [29, 46–50], signifying the complicated and dynamic interactions occurring within microbial communities. Furthermore, the physical orientation of fungal-bacterial biofilms suggests that their assembly and growth likely involves a temporal component [51–55].

Since DFU microbiomes are more complex, comprised of multiple species alongside *C. albicans* and *S. aureus*, and can persist for weeks or months, we hypothesize that during the community assembly and succession process, fungal and bacterial interactions, especially through priority effects (effect of early colonizers on later colonizers [56–58]) and competition, can change the physical and compositional structure of a biofilm community. To address this question, we have developed a simple community of microbes isolated from a single DFU sample with established *C. albicans* colonization [26]. From this sample, *C. albicans* was cultivated alongside *S. aureus* and the Gram-negative bacterium *Citrobacter freundii*. Here, we study community assembly in ex vivo human skin wounds and in vitro biofilm models. We show that ecological interactions, including priority effects and interbacterial competition, shape community structure and pathogenesis.

## Results

### Fungal-bacterial interactions alter biofilm structure and spatial organization within ex vivo human skin wounds

Unlike uniform in vitro *models* utilizing synthetic materials, a human skin ex vivo wound model allows us to investigate biofilm architecture of single, dual, or three-member communities across the spatially structured environment and heterogenous biotic substrate represented by human skin [59–64] using scanning electron microscopy (SEM). With this model, priority effects, or the impact of an early colonizer on a later colonizer within a community [65–67] were tested under three conditions. The first condition represents neutral or no priority, where both microbes are coinoculated simultaneously and incubated for 48 h. Then, priority effects were tested by staggering inoculation, where one partner was given priority and grown for 24 h before the second was inoculated and allowed to grow for an additional 24 h.

**Fig. 1:**
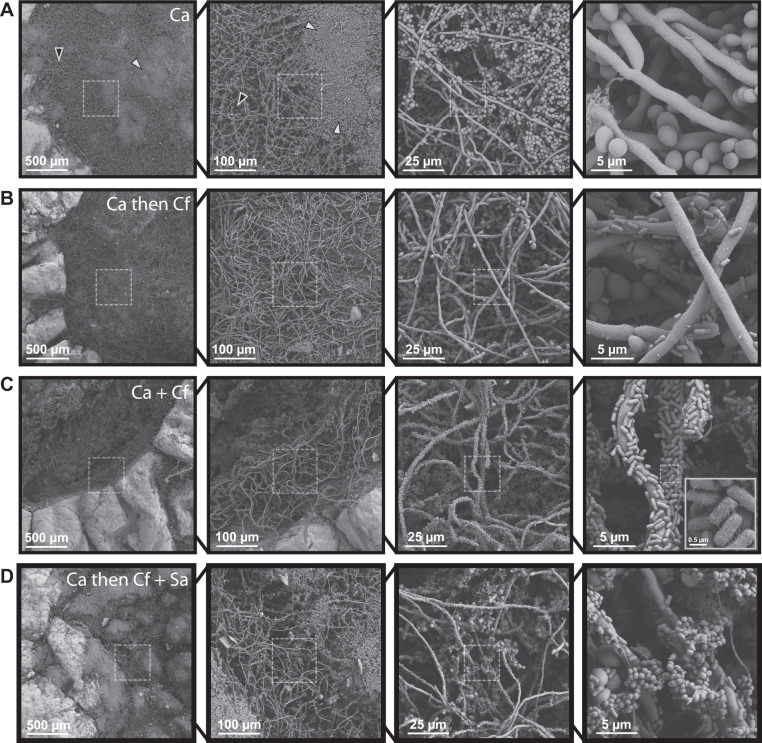
Fungal-bacterial interactions and morphological heterogeneity within wound environments. Scanning electron micrographs of ex vivo wounds at four different magnifications (×100, ×500, ×2000, ×10000). Fungal-bacterial biofilms were grown using both staggered and simultaneous inoculation models in a subset of combinations to illustrate effects of priority and interbacterial competition. Microbes were growth for up to 48 h before SEM processing in 6 mm excisional wounds on 12 mm punch biopsies of human skin suspended in a DMEM-agarose gel at 37 °C, 5% CO_2_. **A** *C. albicans* mono-infection. White arrowheads point to examples of yeast aggregates while black arrowheads point to hyphal networks. **B** *C. albicans* as early colonizer and *C. freundii* as late colonizer**. C** *C. albicans* and *C. freundii* simultaneously coinoculated. For imaging of putative pili on *C. freundii*, magnification was increased to ×20,000 as needed. **D** *C. albicans* as early colonizer and *C. freundii* + *S. aureus* as late colonizers. Dashed outlines represent region magnified.

Human skin was obtained from donors undergoing elective surgery and used to create 6 mm excisional wounds within a 12 mm biopsy of full-thickness tissue. We directly observed physical interactions between *C. albicans*, *C. freundii*, and *S. aureus* with this human ex vivo wound model. *C. albicans* mono-infected wounds were covered with dense aggregates of yeast cells nested among open hyphal networks (Fig. 1A). *C. freundii* mono-infected wounds featured a dense layer of bacteria and small aggregates associated with collagen fibers and extracellular polymeric substances (Fig. S1A). In the *S. aureus* mono-infected wounds, sparse *S. aureus* aggregates adhering to both collagen and aggregated red blood cells were observed (Fig. S1C). We then imaged wounds co-infected with *C. albicans* and *C. freundii* under neutral priority (i.e., coinoculation). Under this condition, the wound bed was covered in extensive *C. albicans* hyphal networks with cells of *C. freundii* substantially attaching to and colonizing the fungal structures, clearly binding to *C. albicans* as opposed to forming clusters in the interstitial space (Fig. 1C). Furthermore, structural features such as putative pili were observed on the surface of individual rod-shaped bacterial cells (Fig. 1C inset). Collagen fibers coated in *C. freundii* were also clearly visible, indicating that both collagen and *C. albicans* are viable substrates for *C. freundii* attachment. In contrast to the *C. albicans* mono-infected wounds, few aggregates of yeast cells were observed (Fig. 1C). Under conditions giving priority to *C. albicans* prior to the addition of *C. freundii*, a similar phenotype was observed. However, fewer aggregates of yeast cells and pseudohyphae were present compared to *C. albicans*-only wounds (Fig. 1B). This suggests that *C. freundii* may trigger the *C. albicans* yeast-to-hyphae phenotypic transition. Conversely, when *C. freundii* had priority over *C. albicans*, no hyphae were observed, and aggregates of *C. albicans* yeasts were seen on dense beds of *C. freundii* (Fig. S1B). As expected based on the literature, when *C. albicans* has priority, we observed *S. aureus* aggregates bound to pre-formed *C. albicans* biofilms (Fig. S1D).

Both *S. aureus* and *C. freundii* are reported to physically attach to *C. albicans* biofilms comprised of yeast and hyphae [26, 38, 40, 68]. We asked if bacteria could coadhere to the fungal scaffold, forming an integrated three-species biofilm. To test this, *C. albicans* biofilms were grown for 24 h, followed by the addition of both bacterial species. We observed few *S. aureus* aggregates adhered to *C. albicans* hyphae and found extensive *C. freundii* colonization and adhesion to both yeast and hyphal forms of *C. albicans* (Fig. 1D), suggesting that *C. freundii* may compete with *S. aureus* to adhere to *C. albicans* biofilms.

### Fungal-bacterial interactions exhibit priority effects in an ex vivo human skin wound model

**Fig. 2:**
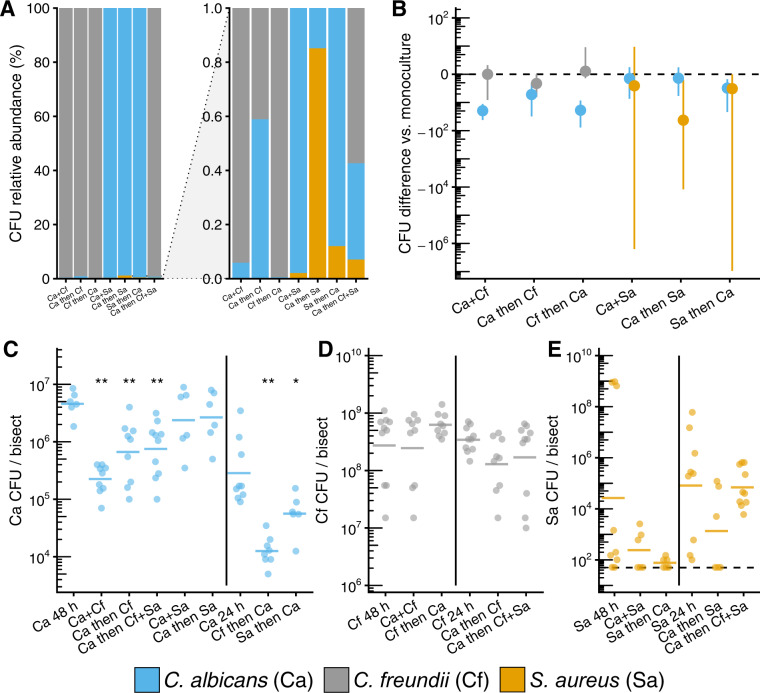
Fungal-bacterial interactions within ex vivo wound model. Fungal-bacterial biofilms grown using both staggered and simultaneous inoculation models. Microbes were grown for up to 48 h in 6 mm excisional wounds on 12 mm punch biopsies of human skin suspended in a DMEM-agarose gel at 37 °C, 5% CO_2_. **A** Relative abundance (full scale and zoomed) of *C. albicans*, *C. freundii*, and *S. aureus* across priority effect models. Stacked bars calculated from means of CFU data shown in (**C**–**E**). **B** Summary of CFU differences between priority effects models and time-matched mono-cultures. Data points show median differences of microbes in co-infections to mono-infections with 95% confidence intervals calculated from CFU data shown in (**C**–**E**) for each microbe using the Mann–Whitney *U* test. Note that the non-parametric confidence intervals are asymmetric around the median and that *C. freundii* and *S. aureus* confidence intervals that do not include 0 are not significantly different due to multiple comparisons corrections. **C** *C. albicans* CFUs across inoculation conditions. **D** *C. freundii* CFUs across inoculation conditions. **E** *S. aureus* CFUs across inoculation conditions. Each data point represents one replicate bisect of a biopsy; horizontal bars show means of ≥6 replicates from ≥2 skin donors. * *p* < 0.05, ** *p* < 0.01.

Viable cell counts were used to quantify absolute abundances and proportional abundance (i.e., community structure) of each microbe within the wound biofilms. We broadly found that priority effects led to increases in the relative abundance of the early colonizer and decreases in the late colonizer. For the *C. albicans–C. freundii* pairing, *C. albicans* proportional abundance increased tenfold from 0.056% within a neutral priority model to 0.59% when given priority to *C. freundii*, but decreased to 0.002% when *C. freundii* had priority (Fig. 2A).

To identify the changes in absolute microbial counts driving these compositional changes, we compared viable cell counts of mixed-cultures to time-matched mono-culture controls. Priority effects favoring a higher overall relative abundance of the early colonizer were the result of a decrease in the absolute abundance of the late colonizer while having neutral or no effect on the early colonizer. Compared to mono-cultures, *C. albicans* had 1.3 log_10_ lower cell counts when *C. freundii* had priority (*p adj*. <0.01) and 0.5 log_10_ lower cell counts when *S. aureus* had priority (*p adj*. < 0.05; Fig. 2B, C). We noted that *C. albicans* had significantly lower absolute abundances under neutral and priority conditions with *C. freundii* (1.3 and 0.7 log_10_ CFU decrease respectively, *p adj*. < 0.01, Fig. 2B, C), which could be due to hyphal induction (Fig. 1A–C). Across all conditions, *C. freundii* and *S. aureus* viable counts were not significantly different from mono-cultures (Fig. 2B, D, E).

For the *C. albicans–S. aureus* pairing, *C. albicans* proportional abundance was 99.98% when neutral, 99.15% when *C. albicans* had priority, and 99.88% when *S. aureus* had priority. Interestingly, *S. aureus* exhibited increased proportional abundance ex vivo when inoculated onto *C. albicans* biofilms; *S. aureus* proportional abundance was 0.017% when neutral, 0.12% when *S. aureus* had priority, and 0.85% when *C. albicans* had priority. Finally, we found that the tri-culture biofilms (0.36% *C. albicans*, 99.58% *C. freundii*, 0.067% *S. aureus*) resembled the composition of biofilms when *C. albicans* is given priority to *C. freundii* (0.59% *C. albicans*, 99.41% *C. freundii*), further suggesting that *C. freundii* competes with *S. aureus*. However, we observed high inter-donor variability in *S. aureus* colonization (Fig. 2E) that could not be explained by inhibition of growth in the tissue culture media (Fig. S2A). We tested *S. aureus* colonization of aged biopsies (5 days post-collection) and found colonization was higher than 1-day-old biopsies from the same donor utilized for our experiments (Fig. S2B). Not unexpectedly, this suggests host-factors in the local tissue environment may influence colonization in this model. Nonetheless, our results demonstrate that priority effects and inter-species competition are important factors influencing community assembly and biofilm architecture.

### Priority effects alter biofilm species composition and growth interactions

**Fig. 3:**
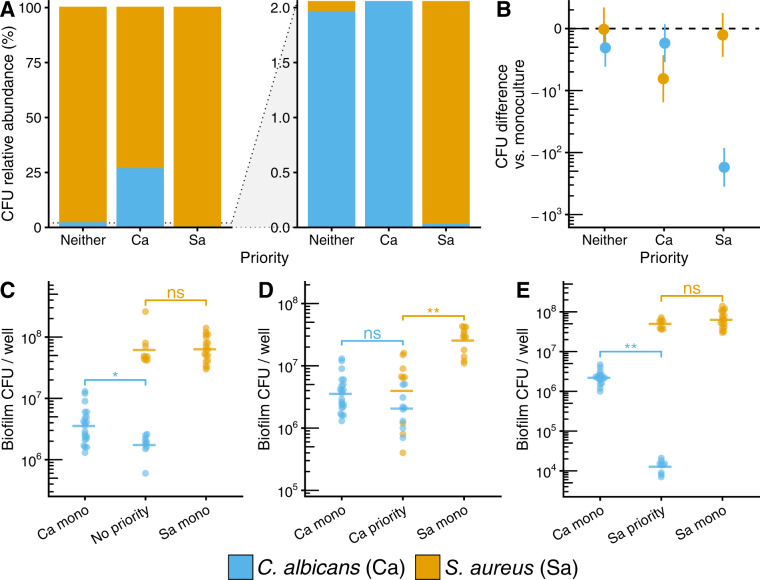
*C. albicans–S. aureus* growth interactions are altered by priority effects. **A** Relative abundance plots (full scale and zoomed) of in vitro *C. albicans*–*S. aureus* biofilms growth in RPMI-1640 media at 37 °C using both staggered and simultaneous inoculation models. Stacked bars represent means of CFU quantification shown in (**C**–**E**). **B** Summary of overall priority effects on mixed-species culture. Co-cultures were subtracted from time-matched mono-culture controls. Data points show mean differences with 95% confidence intervals calculated from CFU data shown in (**C**–**E**) for each microbe using a one-way ANOVA followed by Tukey’s HSD test. Differences are significant if confidence intervals do not include 0. **C** CFUs for *C. albicans–S. aureus* biofilms where *C. albicans* and *S. aureus* were inoculated simultaneously (no priority effect) and grown for 48 h, and time-matched mono-culture controls (48 h). **D** CFUs for *C. albicans–S. aureus* biofilms where *C. albicans* was inoculated 24 h before *S. aureus* (*C. albicans* exerts priority effect) and grown for 48 h, and time-matched mono-culture controls (*C. albicans* 48 h, *S. aureus* 24 h). **E** CFUs for *C. albicans–S. aureus* biofilms where *S. aureus* was inoculated 24 h before *C. albicans* (*S. aureus* exerts priority effect) and grown for 48 h, and time-matched mono-culture controls (*S. aureus* 48 h, *C. albicans* 24 h). For panels (**C**–**E**), each data point represents one replicate well; horizontal bars show means of ≥9 replicates; data are pooled from *n* ≥ 3 independent experiments. * *p* < 0.05, ** *p* < 0.0001, ns: not significantly different.

To follow up on our ex vivo studies and better understand how priority effects impact community composition under controlled conditions, we used an in vitro biofilm model, first evaluating *C. albicans*-*S. aureus* interactions. We found *S. aureus* growth was consistent and reproducible in this model. Under the condition of neutrality, *C. albicans* made up 2.4% of the community, increasing to 26.6% when given priority, and decreasing 1000-fold to 0.026% as a late colonizer. When *C. albicans* is given priority, its absolute abundance is equivalent to a time-matched 48 h mono-culture control. However, as a late colonizer to *S. aureus*, *C. albicans* growth significantly decreased by 2.2 log_10_ relative to the time-matched 24 h mono-culture control (*p adj*. <0.0001; Fig. 3B, D, E). Similarly, *S. aureus* growth as an early colonizer is equivalent to the mono-culture but decreases significantly by 0.81 log_10_ CFU when *C. albicans* has priority (*p adj*. <0.05; Fig. 3B, D, E). When neither partner is given priority, *C. albicans* growth was 0.31 log_10_ CFU lower (*p adj*. <0.05), while *S. aureus* growth was unaffected (Fig. 3B, C). These results demonstrate that although *C. albicans* and *S. aureus* form robust mixed-species biofilm, priority effects can affect overall community composition and alter fungal-bacterial growth dynamics.

**Fig. 4:**
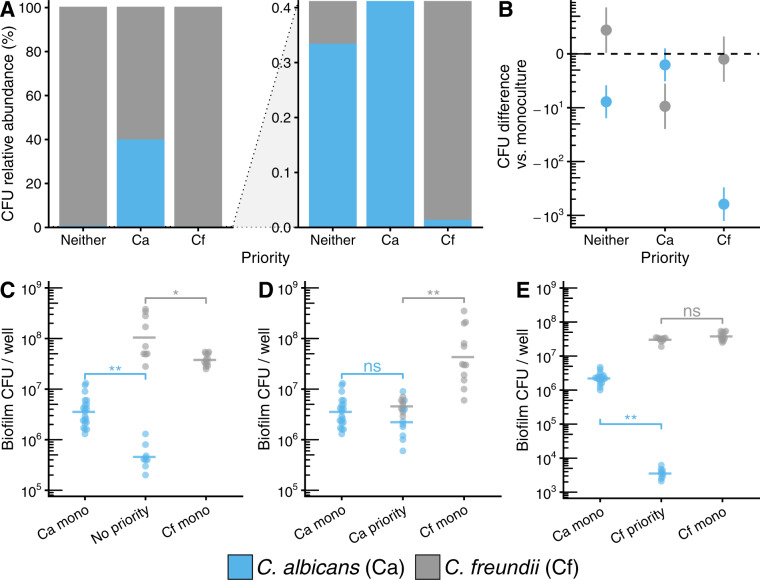
*C. albicans–C. freundii* growth interactions are altered by priority effects. **A** Relative abundance plots (full scale and zoomed) of in vitro *C. albicans*–*C. freundii* biofilms growth in RPMI-1640 media at 37 °C using both staggered and simultaneous inoculation models. Stacked bars calculated from means of CFU data shown in (**C**–**E**). **B** Summary of priority effects on growth in co-culture subtracted from time-matched mono-culture controls. Data points show mean differences with 95% confidence intervals calculated from CFU data shown in (**C**–**E**) for each microbe using a one-way ANOVA followed by Tukey’s HSD test. Differences are significant if confidence intervals do not include 0. **C** CFUs for *C. albicans–C. freundii* biofilms inoculated simultaneously (no priority effect) and grown for 48 h and time-matched mono-culture controls (48 h). **D** CFUs for *C. albicans–C. freundii* biofilms where *C. albicans* was inoculated 24 h before *C. freundii* (*C. albicans* exerts priority effect) and grown for 48 h, and time-matched mono-culture controls (*C. albicans* 48 h, *C. freundii* 24 h). **E** CFUs for *C. albicans–C. freundii* biofilms where *C. freundii* was inoculated 24 h before *C. albicans* (*C. freundii* exerts priority effect) and grown for 48 h, and time-matched mono-culture controls (*C. freundii* 48 h, *C. albicans* 24 h). For panels (**C**–**E**), each data point represents one replicate well; horizontal bars show means of ≥9 replicates; data are pooled from *n* ≥ 3 independent experiments. * *p* < 0.05, ** *p* < 0.0001, ns: not significantly different.

These experiments were repeated with *C. albicans* and *C. freundii*. Similarly, we found that priority effects increased relative abundance of the early colonizer (Fig. 4A), driven by a lower absolute abundance of the late colonizer (Fig. 4B). Within the *C. albicans–C. freundii* pairing, *C. albicans* made up 0.33% of the community under neutral priority with *C. freundii*, and increased to 39.6% with priority, with cell counts matching the mono-culture. As a late colonizer, *C. albicans*’s community proportion decreased to 0.012%, driven by a decrease of 2.8 log_10_ CFU compared to the mono-culture (*p adj*. <0.0001; Fig. 4B, D, E), supporting a competitive exclusion model (Fig. S1B). Under neutral priority conditions, *C. freundii*, made up 99.67% of the community and 99.99% as an early colonizer. However, as a late colonizer, *C. freundii*’s community proportion decreased to 60.4%, due to a 0.9 log_10_ CFU reduction as compared to the mono-culture (*p adj*. <0.0001; Fig. 4B, D, E). As observed with *C. albicans–S. aureus* interactions, priority effects can alter the composition of fungal-bacterial biofilms. Furthermore, we note that low proportional representation in a community (i.e., low relative abundance) does not necessarily correspond to a low absolute abundance. This is especially relevant for *C. albicans*, where a community relative abundance of less than 1% may still equate to an absolute abundance of more than 10^5^ CFUs (Figs. 3, 4).

### *S. aureu*s and *C. freundii* compete for adhesion to C. albicans in mixed-species biofilms

**Fig. 5:**
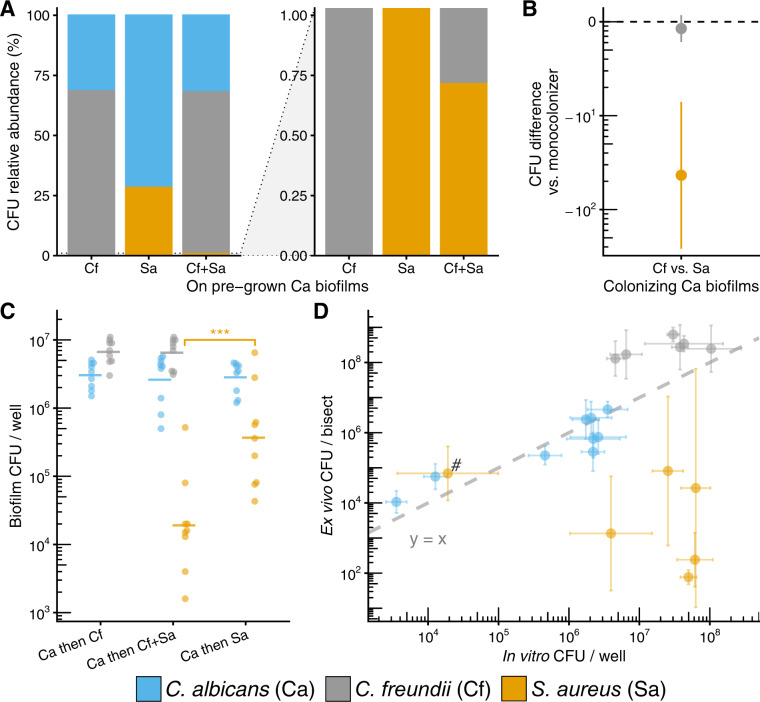
*C. freundii* and *S. aureus* compete when attaching to established *C. albicans* biofilms. **A** Relative abundance plots (full scale and zoomed) of in vitro biofilms in RPMI-1640 media at 37 °C where bacteria (*C. freundii*, *S. aureus*, or *C. freundii* + *S. aureus*) are inoculated onto pre-established 48 h *C. albicans* biofilms and are allowed to grow for an additional 24 h. Stacked bars calculated from means of CFU data shown in (**C**). **B** Summary of interbacterial competition effects on growth on pre-established *C. albicans* biofilms subtracted from mono-colonizer controls. Data points show mean differences with 95% confidence intervals calculated from CFU data shown in (**C**) for each microbe using a one-way ANOVA followed by Tukey’s HSD test. Differences are significant if confidence intervals do not include 0. **C** CFUs for fungal-bacterial biofilms where bacteria (*C. freundii*, *S. aureus*, or *C. freundii* + *S. aureus*) are inoculated onto pre-established *C. albicans* 48 h biofilms. For panel (**C**), each data point represents one replicate well; horizontal bars show means of ≥9 replicates; data are pooled from *n* ≥ 3 independent experiments. * *p* < 0.05, ** *p* < 0.01, *** *p* < 0.001. **D** Correlation plot between CFU counts from ex vivo and in vitro models. Data points are colored by organism for each condition and represent means with error bars showing standard deviation. Dashed line represents line where y = x. # indicates *S. aureus* counts in tri-culture with *C. albicans* and *C. freundii*.

To determine if bacterial competition for attachment sites to the fungal scaffold occurs as suggested by our ex vivo model*, C. albicans* biofilms were grown for 48 h to ensure biofilm maturity, followed by the addition of each bacterial species alone or together, with growth quantified after 24 h. When added alone, *S. aureus* grows to a cell density of 5.6 ± 0.7 (SD) log_10_ CFU/well, representing 28.3% of the community, while *C. freundii* grows to a density of 6.8 ± 0.2 log_10_ CFU/well representing 68.5% of the community. *C. albicans*, as the early colonizer was not affected in growth by late bacterial colonizers (Fig. 5C). When *C. freundii* and *S. aureus* are introduced simultaneously to *C. albicans* biofilms, *S. aureus* growth is reduced by 1.6 log_10_ CFU (*p adj*. <0.001), resulting in an altered community structure comprised of 0.71% *S. aureus* (Fig. 5A–C). Coinoculation with *S. aureus* does not affect the growth of *C. freundii* compared to *C. freundii* alone, offering further support of bacterial competition for fungal attachment sites. Compositionally, the tri-culture biofilms (31.9% *C. albicans*, 67.4% *C. freundii*, 0.71% *S. aureus*) were similar to *C. albicans–C. freundii* biofilms when *C. albicans* has priority (31.5% *C. albicans*, 68.5% *C. freundii*; Fig. 5A). Together, our results demonstrate that priority effects and inter-species competition are important factors influencing community assembly and biofilm formation.

We compared attachment and colonization efficiencies between in vitro conditions and ex vivo wounds and found that *C. freundii* and *C. albicans* colonize the wound tissue at cell densities similar to in vitro. *S. aureus* grew to lower absolute abundances ex vivo as compared to in vitro conditions across both mono- and co-cultures due to high inter-donor variability, except in the tri-culture condition (Figs. 5D, 2E).

### *C. freundii* adheres to *C. albicans* via mannose-specific type I fimbriae, induces hyphae formation, and enhances neutrophil killing

**Fig. 6:**
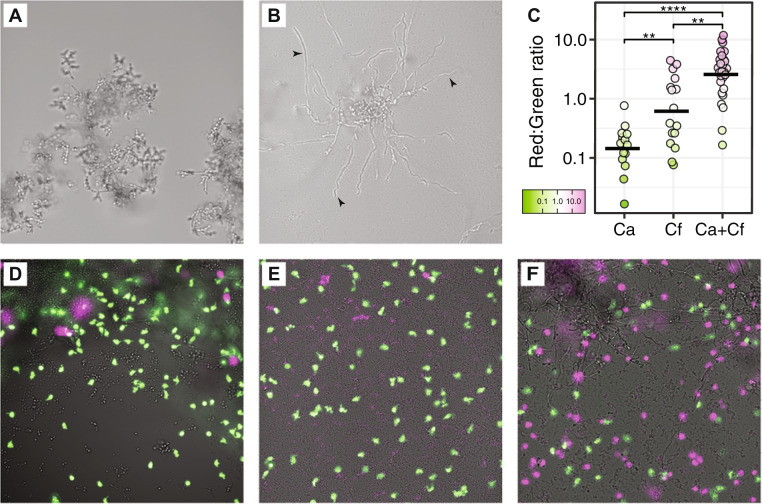
*C. albicans–C. freundii* interactions increase hyphal induction and neutrophil death. Mono- and co-culture biofilms of *C. albicans* and *C. freundii* were grown in RPMI-1640 media for 24 h at 37 °C in chambered coverslips set on a 30° angle to expose the biofilm edge for imaging. *C. albicans* has increased hyphal biofilms when co-cultured with *C. freundii*. **A** *C. albicans* mono-culture biofilm presents a globular phenotype. **B** *C. albicans* and *C. freundii* co-culture biofilm develops long *C. albicans* hyphae. Black arrowheads point to examples of hyphae. Micrographs are representative of at least three independent experiments. **C** Human neutrophils were stained with calcein AM (green) and loaded onto chambered coverslips and allowed to interact with the biofilms for 4 h. Propidium iodide (false-colored magenta) was then added to stain extracellular DNA and cells with permeabilized membranes. Panel shows *C. albicans* mono-culture biofilm (**D**) *C. freundii* mono-culture biofilm (**E**) *C. albicans* and *C. freundii* co-culture biofilm. Micrographs are representative of two independent experiments. **F** Image quantification of red:green fluorescence ratio. A Kruskal-Wallis test followed by pairwise Mann–Whitney *U* tests with the Benjamini-Hochberg correction were used to compare between groups. * *p* < 0.05, ** *p* < 0.01, *** *p* < 0.001, **** *p* < 0.0001.

Our data suggest that *C. freundii* does not inhibit *C. albicans* growth or cause death, as is the case with other Gram-negative bacteria such as *Pseudomonas aeruginosa* and *Acinetobacter baumanii* [47, 69]. Based on our SEM observations, it appears *C. freundii* may alter *C. albicans* biofilm structure and morphology through induction of the yeast-to-hyphae transition. We used a chambered coverslip to permit observation of in vitro biofilms in situ with light microscopy. Similar to the observations in ex vivo wounds, *C. albicans* mono-culture biofilms exhibit a more globular phenotype primarily consisting of yeast cells and pseudohyphae (Fig. 6A). In contrast, *C. albicans* has a marked increase in hyphal growth when co-cultured with *C. freundii* (Fig. 6B). Bacterial species such as *S. aureus* are known to primarily bind *C. albicans* hyphae via protein-protein interactions, such as *C. albicans* Als3p that is primarily expressed in hyphae [38, 68]. Our data show *C. freundii* is able to bind to both yeast and hyphal cells, so we sought to determine the mechanism of *C. freundii* adherence to *C. albicans* cells. *C. freundii* and other members of the Enterobacteriaceae family are known to encode several pili, including type 1 fimbriae that are mannose-specific. Mannose residues exist as a core component of fungal cell wall mannans and mannoproteins, and therefore are present across all morphologies of *C. albicans* [70–72]. Yeast agglutination assays are used to detect and study sugar-specificities of lectin activity such as of type I fimbriae [73–76]. As a proxy for *C. freundii* adhesion to *C. albicans* within our model, we used *C. albicans* yeast agglutination by *C. freundii* cell suspensions to determine that *C. albicans–C. freundii* physical interactions are mannose-sensitive, and that agglutination of *C. albicans* by *C. freundii* can both be inhibited and reversed by mannose but not galactose (Fig. S3). These data support mannose-binding type I fimbriae as the likely mechanism of adhesion between *C. freundii* and *C. albicans*.

The hyphal morphology of *C. albicans*, along with the yeast-to-hyphae transition, are important virulence traits [77–79]. Based on the observation that *C. freundii* appears to promote the switch to *C. albicans* hyphal growth, we hypothesized that this fungal-bacterial interaction could also alter interactions with host cells. To determine if mixed-species biofilms of *C. freundii* and *C. albicans* alter the inflammatory response, we tested neutrophil responses to mono- or mixed-species biofilms.

We exposed calcein-AM-labeled human neutrophils to 24 h biofilms of *C. albicans*, *C. freundii*, and *C. albicans*–*C. freundii* co-cultures for 4 h within a chambered coverslip. We then stained for membrane-permeabilized neutrophils and extracellular DNA as a proxy for neutrophils that have released NETs. We then imaged the neutrophils across each culture condition and quantified the ratio of the area of red (dead) to green (viable) fluorescence. We found a significant increase in fluorescent staining associated with neutrophil death in the co-culture conditions, compared to both mono-culture of *C. albicans* (*p adj*. <0.0001) and *C. freundii* (*p adj*. <0.01; Fig. 6C-F). These results suggest that fungal-bacterial interactions may result in an increased neutrophil death response, leading to pro-inflammatory phenotypes.

## Discussion

The structure of microbial communities in chronic wounds correlates with wound healing outcomes [6, 10, 13, 26, 80, 81]. Here, we utilized a three-member community comprised of a fungal pathogen and two bacterial species isolated from a DFU to probe community assembly and succession under different conditions. We demonstrate that cooperative and competitive species interactions are influenced by temporal factors to shape the overall community structure and pathogenicity. We show that priority effects can significantly alter the compositional structure of biofilm communities of both the well-characterized *C. albicans–S. aureus* pairing and the uncharacterized *C. albicans*–*C. freundii* pairing. We show these effects are consistent across both an in vitro biofilm model as well as an ex vivo human skin wound model. While fungi and bacteria both engage in niche competition for attachment to the underlying substrate such as host tissue or an abiotic surface, bacteria have the additional advantage of being able to colonize fungal cells directly. As a result, interbacterial competition occurs for attachment and colonization to fungal structures. Using SEM, we qualitatively characterized biofilm morphology and spatial organization across ex vivo human skin wounds. We found that *C. freundii* adhesion to *C. albicans* is mediated by mannose-specific binding and triggers *C. albicans* hyphal growth both ex vivo and in vitro. Finally, we showed that the interaction between *C. freundii* and *C. albicans* tunes neutrophil responses leading to increased cell death as compared to mono-cultures. This supports the hypothesis that mixed-species biofilms contribute to enhanced inflammation. Collectively, these results illustrate how competition during community assembly and succession processes can drastically affect community structure and subsequent host response. Our results also underscore the importance of including a temporal lens in studying microbial interactions within wound biofilms.

Physical interactions between fungi and bacteria have been shown to result in enhanced persistence, virulence, and antimicrobial resistance across different disease contexts and environments, including chronic wounds, dental caries, and the cystic fibrosis lung [26, 44, 45, 50, 55, 82–84]. However, microbial communities also change over time through the cyclical process of assembly and succession, especially after disruptions to the community. In the context of chronic wounds, this occurs through standard care procedures such as wound cleansing and mechanical disruption by debridement [81, 85–87]. We found that priority effects alter the microbial community composition between *C. albicans* and two different bacterial species (*C. freundii* and *S. aureus;* Figs. 1–5). Late colonizers experience negative effects with a larger fitness cost to late colonizing *C. albicans*. We hypothesize that this is due to the physical nature of fungal-bacterial interactions. Fungi can be an order of magnitude larger in size than a typical bacterial cell. Thus, fungi like *C. albicans* can play a structural role in providing bacteria with a substrate to attach to, dampening the priority effect. Conversely, bacterial priority effects exclude *C. albicans*, removing potential fungal attachment sites and thereby de-stabilizing the community. When *C. albicans* biofilms are allowed to establish, we find that bacteria compete for adherence to fungal hyphal structures with *C. freundii* outcompeting the professional skin pathogen *S. aureus*. This is an intriguing finding, suggesting community dynamics can limit the expansion of *S. aureus* by competitive exclusion. Over 95% of DFU are colonized by *S. aureus* [13], but not all progress toward a spreading infection such as osteomyelitis or systemic bacterial infection. Our findings may offer insights into interactions suppressing *S. aureus* abundance within the chronic wound environment, which we hypothesize may limit its pathogenicity and virulence. The potential for microbial interactions is high, given the diversity of the wound microbiome. Understanding these interactions is complex as community structure can also be shaped by external factors such as the mechanism of injury, where a blunt trauma may introduce different environmental contaminants than an ulcer forming as the result of anatomical deformities [88].

Although *S. aureus* showed variable growth in ex vivo skin compared in vitro culture conditions, this is expected between different patient samples and the general trends remained consistent (Figs. 1, 2, S1). *Candida albicans* acted as a substrate for *S. aureus* attachment, leading to an increase in its proportional abundance in the ex vivo model. This observation illuminates the challenges translating in vitro data into clinically relevant models. With *S. aureus*, differences in the models may highlight the utility of ex vivo models to study skin-derived innate immunity while isolated from circulating immune cells [60, 89]. We confirmed that media components such as complement proteins from fetal bovine serum in the tissue culture media do not inhibit growth of *S. aureus* (Fig. S2A). We hypothesize that both strain- and donor-specific factors may result in the observed variation in *S. aureus* colonization. Bacterial colonization and adhesion factors, as well as antimicrobial functions of host innate immunity, such as through antimicrobial peptides, have been shown to contribute to variations in *S. aureus* colonization and carriage [90–93]. We note, however, that *S. aureus* colonization within our ex vivo model was most consistent with in vitro results within the tri-culture condition, showing increased colonization and decreased variability compared to mono- and co-culture conditions (Fig. 5D, data point marked by #), indicating that polymicrobial interactions may add to the complexity of host-microbe colonization dynamics.

We observed that *C. freundii* attaches to yeast, pseudohyphal, and hyphal forms of *C. albicans*. In contrast, *S. aureus*, *P. aeruginosa*, and *A. baumanii* have all been reported to preferentially adhere to *C. albicans* hyphae [38, 47, 69]. This may account for the ability of *C. freundii* to effectively outcompete *S. aureus* for binding if *C. albicans* biofilms of clinical isolates are phenotypically heterogenous [94]. Furthermore, we found that *C. freundii* attachment to *C. albicans* cells induce hyphae formation, leading to more surface area as hyphae expand across the tissue surface to create a tangled three-dimensional network. We observed cellular appendages on *C. freundii* that appear to mediate the adhesion to *C. albicans* (Fig. 1C inset) and hypothesize that these appendages are type I pili, consistent with our finding that *C. freundii* induced agglutination of *C. albicans* yeasts can be both inhibited and reversed by mannose (Fig. S3). Mannose residues are a key component of the fungal cell wall regardless of morphology [95] and mannose-binding type I fimbriae are ubiquitous among the species in the Enterobacteriaceae family, such as *C. freundii* [75, 96]. Furthermore, persistence of bacteria in the Enterobacteriaceae family has been reported as a microbial marker and predictor of poor wound healing in DFUs localized to the heel of the foot [10].

We used viable cell enumeration on selective plates to quantify growth within biofilms. A limitation of this technique is that adhesive cell clusters and fungal hyphae, while functionally different compared to a single cell, will also plate as one countable colony. Although we found that *C. albicans* CFUs were reduced when in co-culture with *C. freundii* (Figs. 2, 4), the magnitude of change may not be absolute because of induced morphological differences in *C. albicans* due to *C. freundii* (Figs. 1, 6A, B). Fungal hyphae in general are difficult to quantify [97]. However, we want to note that our viable counts were consistent and reproducible. Furthermore, our use of SEM to investigate the spatial structure of biofilms supports our observations of reduced *C. albicans* cell counts due to hyphal induction, such as during *C. albicans* and *C. freundii* co-colonization. These observations raise the question of what might be missed if we study pairwise interactions by quantifying absolute abundances. Morphological changes in *C. albicans* may have a far greater impact on virulence than overall viable cell counts [77, 98–100]. We reiterate that proportional representation in a community (i.e., relative abundance) does not necessarily correspond to absolute abundance, and furthermore cannot capture physical and functional characteristics of the resulting community [101].

We found that *C. albicans* and *C. freundii* mixed biofilms increase neutrophil death. Neutrophils are among the first responders during the inflammatory phase of wound healing. Their primary role is to clean the wound of debris and contaminating microbes through phagocytosis. Neutrophils also undergo a cell death process to release neutrophil extracellular traps (NETs), which are web-like structures of DNA and antimicrobial proteins that trap and kill microbes to prevent them from spreading. Mono-cultures of *C. albicans* biofilms have been shown to inhibit NETosis [102] but the increased length of hyphae compared to yeast cells increases incomplete or “frustrated” phagocytosis and also alters reactive oxygen signaling in the neutrophil response [78, 103]. Attachment of *C. freundii* induces a morphological change in *C. albicans* and results in increased hyphae and neutrophil cell death. In the context of diabetes, neutrophils in both diabetic mice models and diabetic patients are primed to undergo NETosis [104, 105]. It is thought that the increased inflammation triggered by NETosis likely contributes to the delayed wound healing associated with this disease. Thus, we hypothesize that a subset of neutrophil-associated inflammation may be due to fungal-bacterial interactions.

The presence of pathogenic fungi in wounds is correlated to poorer wound healing outcomes and can complicate treatment [26–28]. A major component of the chronic wound microbiome is community stability, or the lack of, as being a key factor for predicting wound outcomes [6, 14]. Fungi such as *C. albicans*, although representing a small proportion of a community, provide a scaffold to colonizing bacterial species and contribute to overall community stability. We have shown that this process is affected by ecological factors such as order of arrival to a community and subsequent priority effects can drastically alter the physical and compositional structure of biofilm communities. In turn, virulence traits and host responses are altered. We anticipate as we uncover more ecological principles relevant to microbial growth and biofilm formation within wounds, a combination of bottom-up analyses building complexity within our models and top-down approaches such as metatranscriptomics will add to our understanding of the microbial impact on wound healing with positive implications for future basic and translation research.

## Materials and methods

### Strains and culture conditions

#### (1) Fungal-bacterial biofilms in 96-well plates

Isolates were grown overnight at 37 °C on yeast extract-peptone-dextrose (YPD; *C. albicans*) or tryptone soy (bacteria) agar plates. Inoculums were made by suspending colonies into sterile PBS followed by dilution in RPMI-1640 with 2% glucose and 0.165 M MOPS (pH 7.0) to a final cell density of 1 × 10^5^ CFU/mL. Biofilms were grown statically at 37 °C in non-treated polystyrene 96-well plates (CC7672-7596; USA Scientific, Ocala, FL). For mono-cultures, 200 μL of inoculum was added to each well and incubated for 24 h or 48 h with fresh media replaced at 24 h. For staggered inoculation, 200 μL of inoculum containing the early colonizer was added to each well and grown statically for 24 h (or 48 h for competitive bacterial binding to *C. albicans* biofilms). The supernatant was gently removed and 200 μL of inoculum of the late colonizer was added to each well and grown statically for another 24 h. To harvest biofilms, media was removed and each well washed with 2 ×200 μL PBS to remove non-adherent cells. Biofilms were resuspended in 200 μL of PBS before serial dilution and spot plating 20 μL for CFU counts on selective/differential media: YPD agar with 50 μg/mL kanamycin (*C. albicans*), TSA with 50 μg/mL nystatin (*C. freundii* and *S. aureus*), and TSA with 7.5% NaCl and 50 μg/mL nystatin (*S. aureus* in tri-culture).

#### (2) Ex vivo human skin wound model

Human skin was obtained from patients undergoing elective reconstructive surgeries. The deidentified samples were exempt from the regulation of University of Wisconsin-Madison Human Subjects Committee Institutional Review Boards. The tissue was rinsed with PBS and partial-thickness wounds were made by puncturing the epidermis with a 6 mm biopsy punch and removing the entire epidermis and a portion of the dermis. A 12 mm biopsy punch was then used to make full-thickness biopsies with the wound. Biopsies were placed into 12-well plates containing 3 mL of a DMEM-agarose gel (0.15:0.85 ratio of 1% agarose in PBS and Dulbecco’s modified Eagle medium [DMEM] supplemented with 10% fetal bovine serum [FBS]). Biopsies were incubated at 37 °C with 5% CO_2_ and transferred onto new media every 48 h. Inoculums were prepared as described above and diluted to a cell density of 1 ×10^7^ CFU/mL each. Wounds were inoculated within 24 to 48 h of tissue collection for a final cell density of 1 ×10^5^ CFU/wound. For staggered colonization, late colonizers were inoculated as described above, 24 h after inoculation of the early colonizer. Following incubation, biopsies were processed for SEM (see below) or bisected and processed for viable cell enumeration. Bisects were vortexed in 1 mL PBS with 0.2 g of 1 mm sterile glass beads for 10 m at full-speed on a Vortex-Genie 2 (Scientific Industries, Bohemia, NY) before serial dilution and spot plating as described above. To test effect of biopsy age on *S. aureus* colonization, a subset of biopsies were kept sterile and uninfected for 5 days before infection. Donor skin tissue is pre-treated with antiseptics prior to collection during surgery and has been independently confirmed using qPCR and viable cell counts to have no detectable microbial load (data not shown). For each experiment, negative (uninfected) controls were maintained and transferred onto new media every 48 h to monitor for contamination.

#### (3) Growth curves

*S. aureus* inoculums were prepared as described above. Media tested include RPMI-1640 with 2% glucose and 0.165 M MOPS (pH 7.0) and DMEM. Aliquots of FBS were heat-inactivated for 30 m at 56 °C for a subset of media conditions and added to the media at 10% (v/v). Liquid cultures were grown statically at 37 °C in non-treated polystyrene 96-well plates (CC7672-7596; USA Scientific, Ocala, FL) with OD_600_ readings taken every 15 m over 24 h of growth on a plate reader (EPOCH2, Biotek, Winooski, VT).

### Scanning electron microscopy

The following protocol was adapted from ref [106]. Briefly, ex vivo human skin wounds were rinsed with PBS and fixed overnight in 5 mL of 1.5% glutaraldehyde in 0.1 M sodium phosphate buffer (pH 7.2) at 4 °C. Samples were rinsed, treated with 1% osmium tetroxide for 1 h, and then washed again. Samples were dehydrated through a series of ethanol washes (30–100%) followed by critical point drying (14 exchanges on low speed) and were subsequently mounted on aluminum stubs with a carbon adhesive tab and carbon paint. Silver paint was applied around the perimeter for improved conductivity. Samples were left to dry in a desiccator overnight. Following sputter coating with platinum to a thickness of 20 nm, samples were imaged in a scanning electron microscope (Zeiss LEO 1530-VP) at 3 kV.

### Yeast-agglutination assay with sugar competition

Inoculums of *C. albicans* and *C. freundii* were made as described above. 1:10 dilutions of the suspensions were used to quantify OD_600nm_ of 1.0~1.5, corresponding to an undiluted OD_600nm_ of 10~15. To induce agglutination, 100 μL of each microbe and 100 μL of PBS was added to a 1.5 mL microcentrifuge tube and shaken at 175 rpm in a 37 °C incubator for 15 m. To test for inhibition of agglutination, 500 mM D-mannose or D-galactose in PBS was added. To test for reversal of agglutination, 50 μL of PBS, 500 mM D-mannose or D-galactose in PBS was added to 50 μL of the agglutinated *C. albicans* and *C. freundii* suspension in PBS and briefly vortexed to mix. Mono-culture controls were 100 μL of cell suspensions and 200 μL of PBS. Images were take using on a Nikon Eclipse E600 microscope equipped with a Leica DFC420 camera using LAS v4.12 software. Objective used was the Nikon Plan Fluor 40x using the Ph2 annulus on the sub-stage condenser.

### Fluorescence imaging of neutrophil interactions in vitro

Human neutrophils were collected as previously described [102]. For fluorescent imaging, the following protocol was adapted from ref. 102. In total, 100 μL of fungal and bacterial cells in RPMI-1640 (1 × 10^5^ cells/mL) were loaded into the wells of a tissue culture-treated μ-Slide (8 wells, ibidi, Fitchburg, WI) and grown on a 30° degree angle using a well plate stand for 24 h at 37 °C with 5% CO_2_. Neutrophils, stained with calcein AM (Thermo Fisher Scientific, Waltham, MA) at 0.5 µg/ml in DPBS for 10 m at room temperature in the dark, were added at a concentration of 1 × 10^5^ cells/well and allowed to incubate flat for 4 h at 37 °C with 5% CO_2_. Propidium iodide (3 µM) incubated with samples for 15 m at 37 °C was used to visualize extracellular DNA and neutrophils with disrupted membranes. Images were obtained with a Nikon eclipse-TI2 inverted microscope and ORCA-Flash 4.0 LT sCMOS camera using NIS elements imaging software on bright field, FITC, and TexasRed channels using a 20x objective. Images were taken from random fields of view along the biofilm leading edge. Exposure times and linear contrast (LUTs) for each channel were fixed and consistent within each independent biological experiment. Image channels were exported separately and analyzed using FIJI [107]. Single channel images were converted to grayscale and the Auto Threshold function using the IJ-IsoData algorithm on a dark background was used to identify neutrophils. The pixel area occupied by neutrophils was calculated for each channel separately and the percent area of the red channel divided by the green channel was reported as the red:green ratio to normalize for varying amounts of neutrophils in each field of view. Brightfield, FITC, and TexasRed channels were overlaid for display within Fig. 6, with additional modifications including manual adjustment of linear contrast and conversion of the red channel to magenta for visualization and accessibility purposes.

### Data and statistical analysis

Information regarding sample size and replication are described in the figure legends. All statistical analysis was performed using R [108]. Multiple comparisons and estimation of mean (parametric) or median (non-parametric) differences between inoculation conditions for each microbe were evaluated using a one-way between subjects ANOVA with Tukey’s Honest Significant Differences test or the Kruskal-Wallis test with pairwise Mann–Whitney *U* tests with the Benjamini-Hochberg correction for normally or non-normally distributed data, respectively. We used an *α* level of 0.05 for all statistical tests.

## Supplementary information

Supplemental Figure Legends

Figure S1

Figure S2

Figure S3
