# Supplementary material for: Priority effects dictate community structure and alter virulence of fungal-bacterial biofilms
Source: ISME J. 2021 Feb 8;15(7):2012–27. doi: 10.1038/s41396-021-00901-5 (PMC8245565; doi:10.1038/s41396-021-00901-5)
Supplement: Supplementary file 1 — Supplemental Figure Legends [file 41396_2021_901_MOESM1_ESM.docx]

## Supplementary Figure 1. *C. albicans* is competitively excluded from *C. freundii* biofilms while *S. aureus* adheres to *C. albicans* biofilms.

Scanning electron micrographs of *ex vivo* wounds at four different magnifications (100x, 500x, 2 000x, 10 000x). Fungal-bacterial biofilms were grown using both staggered and simultaneous inoculation models in a subset of combinations to illustrate effects of priority and interbacterial competition. Microbes were growth for up to 48 h before SEM processing in 6 mm excisional wounds on 12 mm punch biopsies of human skin suspended in a DMEM-agarose gel at 37°C, 5% CO_2_. **A)** *C. freundii* mono-infection. **B)** *C. freundii* as early colonizer and *C. albicans* as late colonizer **C)** *S. aureus* mono-infection **D)** *C. albicans* as early colonizer and *S. aureus* as late colonizer. Dashed outlines represent region magnified.

## Supplementary Figure 2. *S. aureus* is not inhibited by media components and colonization is increased on older biopsies from the same skin donor.

**A)** Growth curves of *S. aureus* in 200 μL of media in a 96-well plate incubated statically at 37°C. Growth curves are shown as averages of 3 replicate wells using generalized additive model smoothing. **B)** CFUs for mono-culture *S. aureus* biofilms grown for up to 48 h in 6 mm excisional wounds on 12 mm punch biopsies of human skin suspended in a DMEM-agarose gel at 37°C, 5% CO_2_. Each data point represents one replicate bisect of a biopsy; horizontal bars show means of ≥ 2 replicates from a single skin donor.

## Supplementary Figure 3. *C. freundii*-induced *C. albicans* agglutination is mannose-sensitive.

Dense suspensions (OD_600nm_ ~ 10-15) of *C. albicans* and *C. freundii* in PBS were combined with PBS, 500 mM D-mannose, or D-galactose in a 1:1:1 ratio and shaken at 175 rpm for 15 m to induce agglutination. For reversal, agglutinated *C. albicans* and *C. freundii* were combined with 500 mM D-mannose or D-galactose in a 1:1 ratio and vortexed to mix. Phase contrast micrographs of fungal-bacterial suspensions at 40x magnification showing agglutination of *C. albicans* yeast cells by *C. freundii* that can be both inhibited and reversed by the addition of mannose but not galactose. Black arrowheads point to examples of agglutinated *C. albicans* clusters.
