# Supplementary figures and images for: Priority effects dictate community structure and alter virulence of fungal-bacterial biofilms
Source: ISME J. 2021 Feb 8;15(7):2012–27. doi: 10.1038/s41396-021-00901-5 (PMC8245565; doi:10.1038/s41396-021-00901-5)

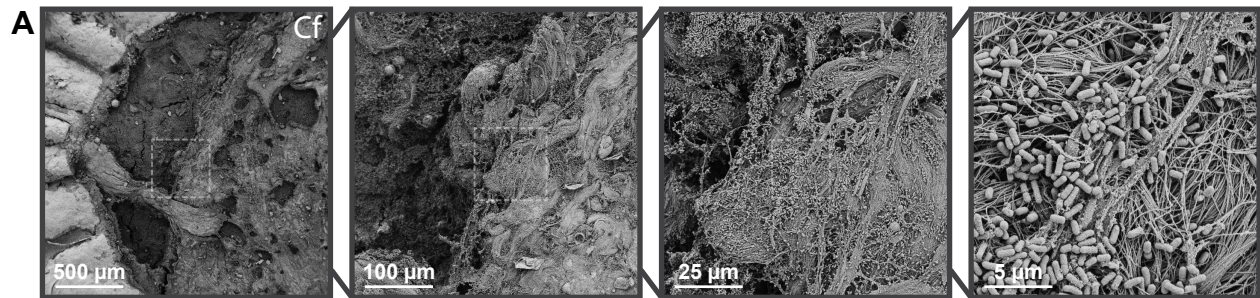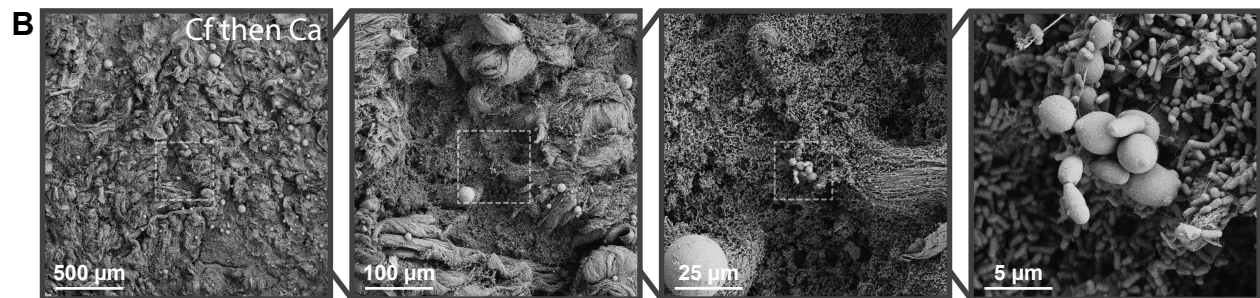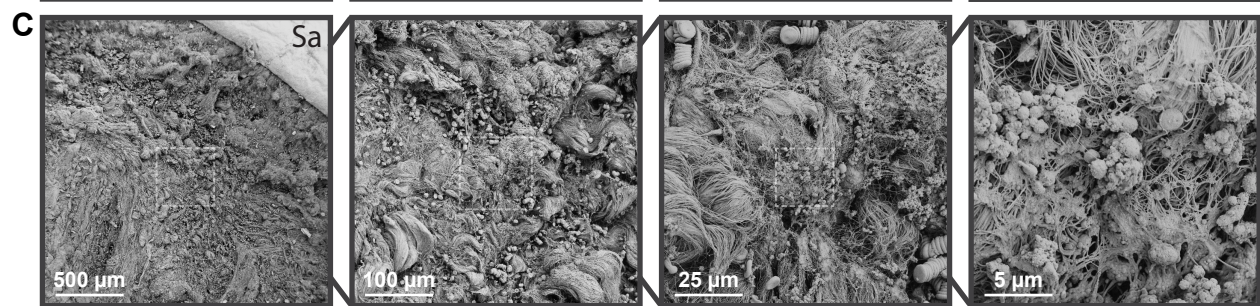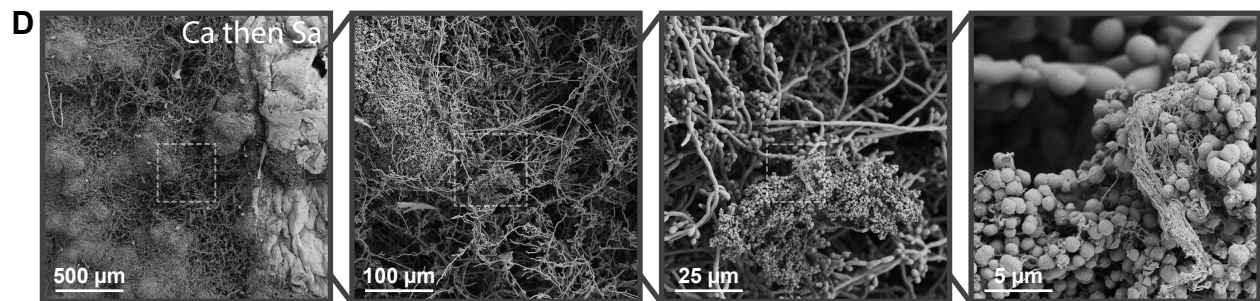

Supplement: Supplementary file 2 — Figure S1 [file 41396_2021_901_MOESM2_ESM.pdf]

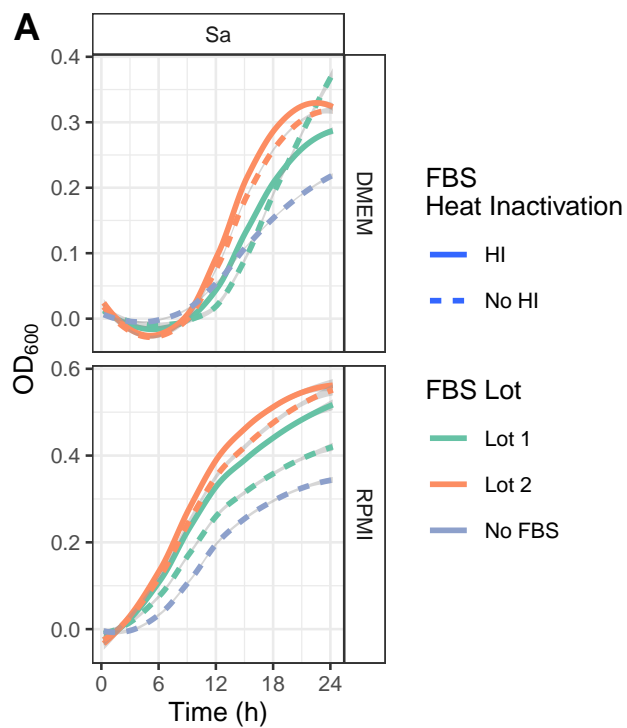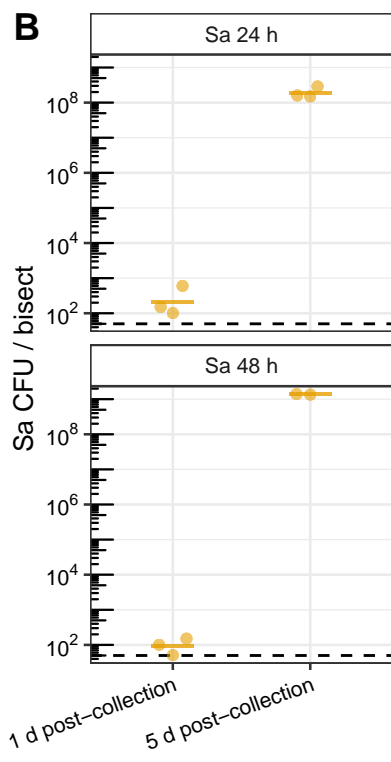

Supplement: Supplementary file 3 — Figure S2 [file 41396_2021_901_MOESM3_ESM.pdf]

**Initial**

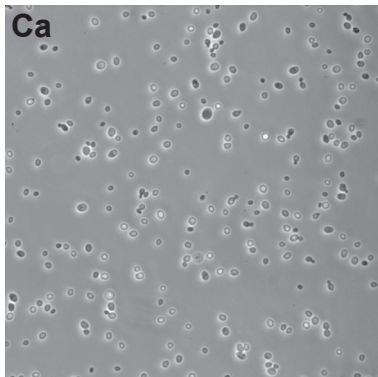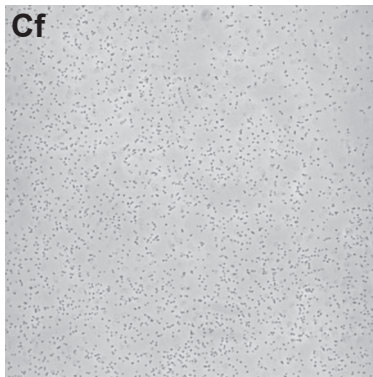

**Inhibition**

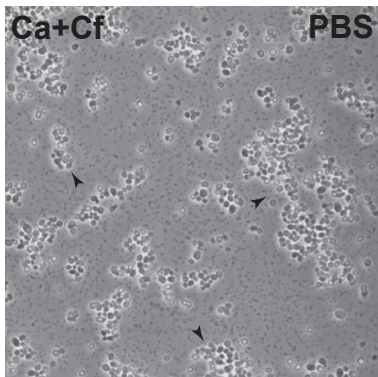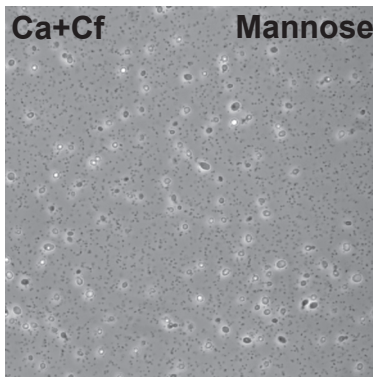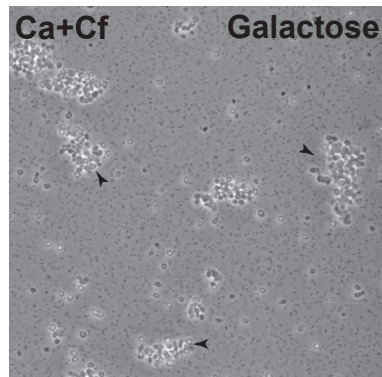

**Reversal**

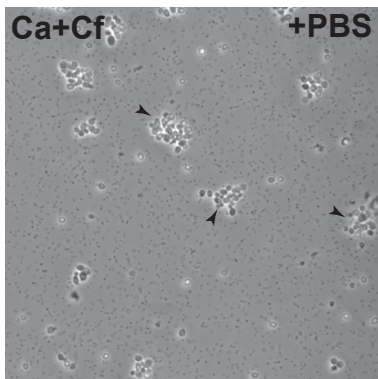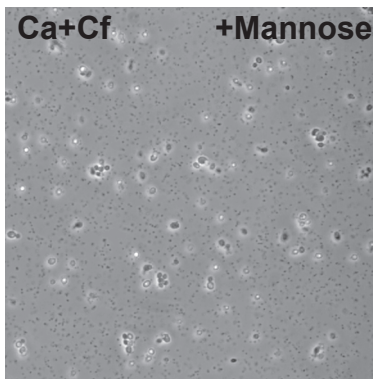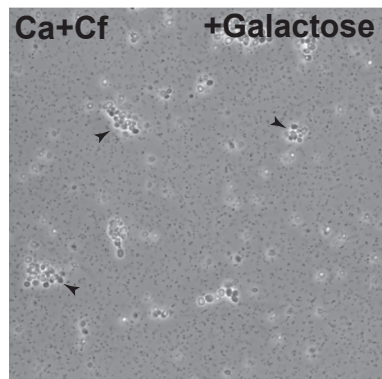

Supplement: Supplementary file 4 — Figure S3 [file 41396_2021_901_MOESM4_ESM.pdf]
